# Understanding the outcomes of COVID-19 – does the current model of an acute respiratory infection really fit?

**DOI:** 10.1099/jgv.0.001545

**Published:** 2020-12-17

**Authors:** Peter Simmonds, Sarah Williams, Heli Harvala

**Affiliations:** ^1^​ Nuffield Department of Medicine, University of Oxford, Oxford, UK; ^2^​ National Microbiology Services, NHS Blood and Transplant, London, UK

**Keywords:** COVID-19, SARS-CoV-2, coronavirus, persistence, respiratory syncytial virus influenza A virus

## Abstract

Although coronavirus disease 2019 (COVID-19) is regarded as an acute, resolving infection followed by the development of protective immunity, recent systematic literature review documents evidence for often highly prolonged shedding of severe acute respiratory syndrome coronavirus 2 (SARS-CoV-2) in respiratory and faecal samples, periodic recurrence of PCR positivity in a substantial proportion of individuals and increasingly documented instances of reinfection associated with a lack of protective immunity. This pattern of infection is quite distinct from the acute/resolving nature of other human pathogenic respiratory viruses, such as influenza A virus and respiratory syncytial virus. Prolonged shedding of SARS-CoV-2 furthermore occurs irrespective of disease severity or development of virus-neutralizing antibodies. SARS-CoV-2 possesses an intensely structured RNA genome, an attribute shared with other human and veterinary coronaviruses and with other mammalian RNA viruses such as hepatitis C virus. These are capable of long-term persistence, possibly through poorly understood RNA structure-mediated effects on innate and adaptive host immune responses. The assumption that resolution of COVID-19 and the appearance of anti-SARS-CoV-2 IgG antibodies represents virus clearance and protection from reinfection, implicit for example in the susceptible–infected–recovered (SIR) model used for epidemic prediction, should be rigorously re-evaluated.

## The acute infection model

Based on its initial association with the respiratory disease termed coronavirus disease 2019 (COVID-19), infections with severe acute respiratory syndrome coronavirus type 2 (SARS-CoV-2) were naturally equated to those of other respiratory viruses – an acute phase characterized by varying degrees of respiratory symptoms, transient high-level virus replication in the respiratory tract followed by virus clearance. Resolution is associated with the appearance of activated T cells that destroy infected cells and local and humoral antibody responses that contain and prevent virus spread. As with influenza A virus (IAV) and respiratory syncytial virus (RSV), potent adaptive immune responses may create a degree of immunopathology associated with excessive reactivity (‘cytokine storms’) in the lungs of some patients. Essentially, however, it is the virus that is pathogenic and its immune-mediated clearance leads to resolution of disease and the development of long-term immunity from reinfection and disease development. The application of molecular and serological diagnostics for SARS-CoV-2 is predicated by this model, as is the widespread use of a simple susceptible–infected–recovered (SIR) infection model to predict the course of the COVID-19 pandemic.

**Fig. 1.**
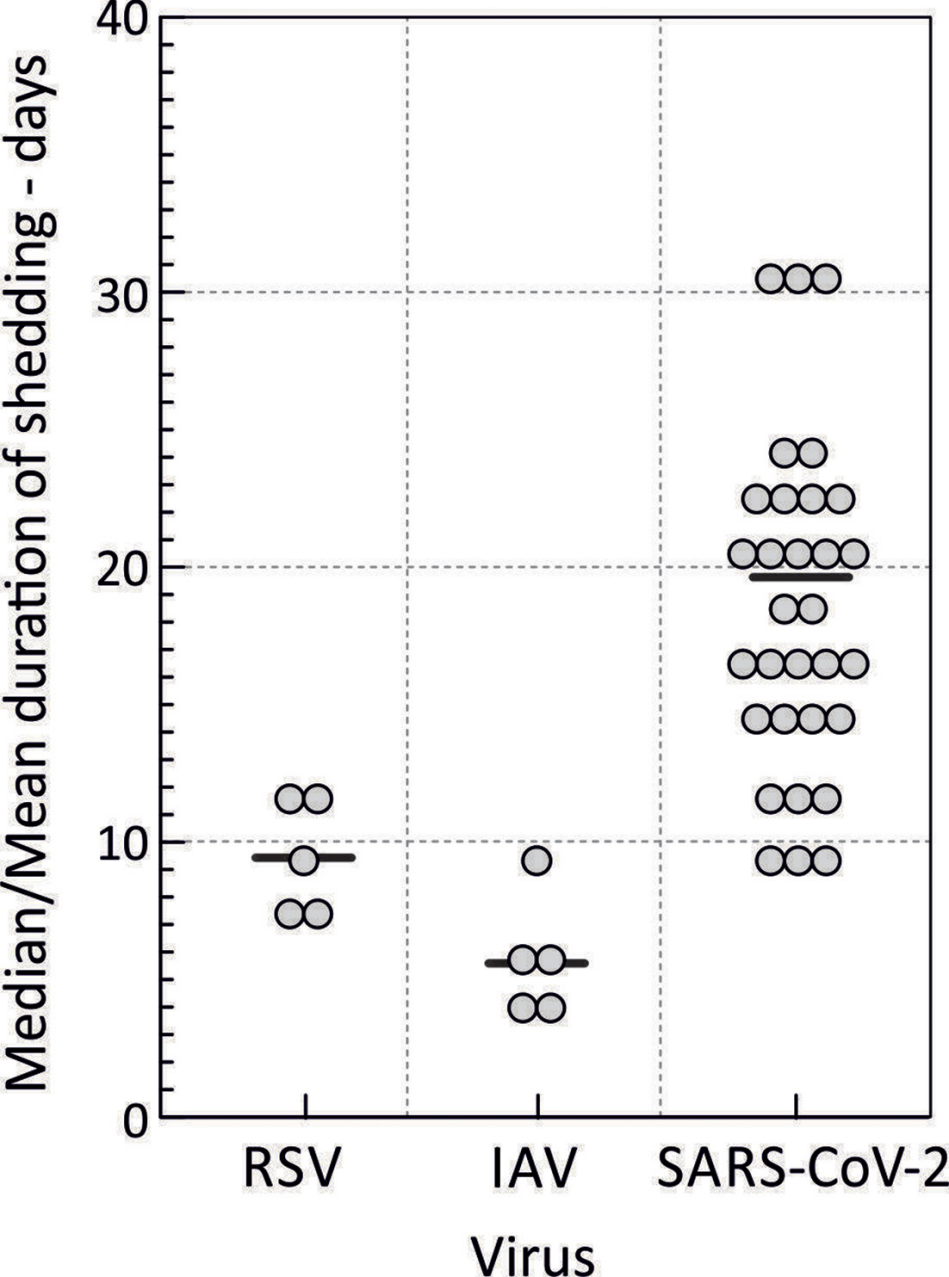
Distribution of mean and maximum recorded shedding periods for SARS-CoV-2 and other respiratory viruses after disease onset. Mean durations of SARs-CoV-2 shedding by PCR in 31 published larger cohort studies (total subjects=4150) [2, 37, 90–97] compared to those of RSV and IAV [1, 7–14]. Solid black lines indicate the mean value of all studies for each virus.

While an acute, resolving pattern of infection is frequently observed, an analysis of cohort studies and case reports investigating durations of shedding of SARS-CoV-2 from the respiratory tract casts severe doubt on the broader applicability of this model. Published reviews report mean durations of SARS-CoV-2 shedding for acute infection of 17–18 days (Fig. 1) [1, 2], and 20 days in severe cases [2], with numerous individual case reports of immunocompetent subjects remaining PCR-positive for longer than 50 days, and in some cases shedding virus for up to 104 days [3–6]. These durations of shedding are systematically and substantially longer than the mean shedding durations of 9.5 and 5.7 days determined by PCR-based follow-up of immunocompetent adults and children infected with RSV [1, 7–10] and IAV [1, 11–14], respectively. Similarly limited durations of shedding have been observed in other pathogenic human respiratory RNA viral pathogens [1].

**Fig. 2.**
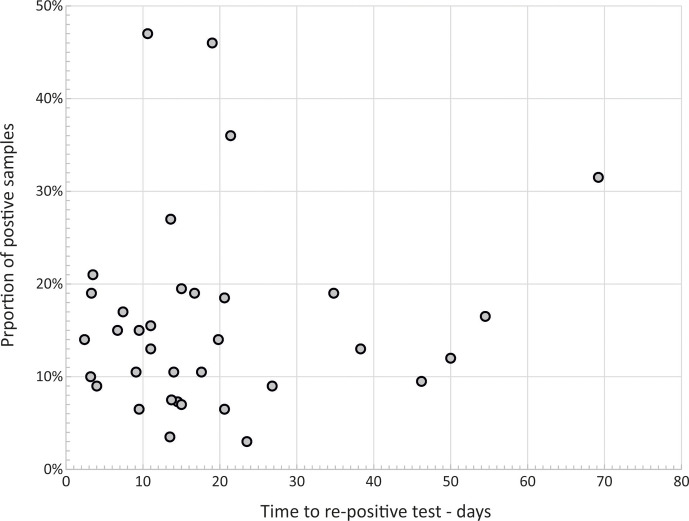
Frequencies of reactivation and time to a re-positive test. Detection frequency of SARS-CoV-2 RNA after two negative tests post-COVID-19. Data points represent the summary data from 35 studies [15–20]; *x*-axis values represent midpoint values for data reported in ranges.

What further sets SARS-CoV-2 apart from other respiratory virus infections is the remarkably frequent reactivation of virus shedding post-COVID-19, defined as SARS-CoV-2 RNA detection after typically two negative tests following resolution of the initial infection (Fig. 2) [15–20]. Detection frequencies initially decline with sampling interval, but several studies document frequent reactivation after 50 days or longer. There is the additional possibility that they may represent reinfections. While the genetic strains in early and late samples were not compared in these earlier studies, numerous instances of reinfection of immunocompetent individuals by different clades of SARS-CoV2 have been now documented [21–26]. Prolonged, reactivated or reinfections all occurred irrespective of the presence of neutralizing antibody to SARS-CoV-2.

Collectively, these observations indicate that virus clearance is not achieved in a proportion of individuals infected with SARS-CoV-2, a phenomenon quite different from what is typically imagined as outcomes of other respiratory RNA virus infections. The upper bound for prolonged infections remains largely undetermined, and can be difficult to fully evaluate – shedding may be intermittent, most studies are based on relatively short follow-up periods dictated by the recent nature of the COVID-19 pandemic, and sites of persistence may be inaccessible for conventional sampling. This is exemplified by the recent discovery of SARS-CoV-2 RNA, nucleoprotein expression and visualization of abundant coronavirus particles by electron microscopy in intestinal biopsies from 5 from 14 individuals infected previously (mean 4 months) with SARS-CoV-2 [27]. These high rates of detection were recorded in individuals who were uniformly negative on conventional respiratory sampling and PCR testing, and suggests that longer-term systemic persistence may be far more frequent that recorded by conventional respiratory sampling in the previously cited studies (Figs 1 and 2). The evident propensity of SARS-CoV-2 to infect non-respiratory tissues again sets it apart from outcomes of typical respiratory virus infections.

### Characteristics of prolonged SARS-CoV-2 infections

Individuals in the majority of the cited studies with prolonged or reactivated infections were not overtly immunosuppressed. Furthermore, the occurrence of prolonged infections was unrelated to the severity of COVID-19; for example, of the 99 from 851 individuals showing prolonged infections, 61 were non-hospitalized during initial infection [28]. The presence of neutralizing antibodies did not appear to substantially influence the prolongation of infections or the recurrence of PCR positivity, but at least two studies demonstrate proteomic or immunological evidence for altered host responses to infection that may influence clearance [29, 30]. It could be argued that the greater durations of shedding of SARS-CoV-2 compared to other respiratory viruses may be the results of differences in past exposure; infections with SARS-CoV-2 are almost invariably the first encounter with the virus, whereas IAV, RSV and other respiratory infections may typically occur in individuals with partial immunity from previous infections that may lead to an attenuated course of replication and virus shedding. However, the occurrence and frequency of prolonged and periodic activation (collectively documented in ~15 % of studied individuals) are nevertheless quite distinct from the almost universal clearance of IAV and RSV infections and establishment of durable immunity. Although data are currently limited, reinfections with SARS-CoV-2 can be as pathogenic and prolonged as primary infections, indicating that even recent SARS-CoV-2 infections may fail to produce the attenuation of disease severity of the kind proposed for IAV and RSV.

In documenting the phenomenon of potential persistence, a further variable is the nature of the samples tested; rates of SARS-CoV-2 PCR positivity were higher in deep respiratory samples late in infection [31], and SARS-CoV-2 was detected in intestinal biopsies substantially beyond the period of respiratory virus shedding [27], an observation that is consistent with observation that faecal excretion typically persists longer than respiratory tract detection (reviewed in [32]). In relation to the latter, screening of sewage or wastewater has been used to monitor circulation of SARS-CoV-2 in the community, similarly to poliovirus and enteric pathogens such as norovirus. However, although there is a strong temporal association between SARS-CoV-2 viral loads in sewage or wastewater and incidence of SARS-CoV-2 infections in the community in early outbreak stages [33, 34], longer-term monitoring through periods of lockdown and after cessation of SARS-CoV-2 circulation showed continued levels or less than proportionate reductions in SARS-CoV-2 detection [35, 36]. One interpretation of these otherwise unanticipated findings is that a substantial number of long-term faecal excretors may exists in the community with undiagnosed persistent infections.

The relationship between prolonged or recurrent positivity in respiratory or gastrointestinal tracts in a proportion of individuals and their infectivity has been widely discussed but is currently unresolved. Prolonged detection of SARS-CoV-2 might arise through residual, non-replicative viral particles and debris from infected cells, and the instances of prolonged or intermittent RNA detection in respiratory samples may not be relevant for onward transmission of the virus. Supporting this, several studies have shown that SARS-CoV-2 cannot be isolated from samples taken 6–8 or more days after COVID-19 development ([37,38, 39]). Against this conclusion, however, is the likelihood that SARS-CoV-2 may indeed be actively replicating but may not be isolatable because of the lower viral loads virus typically found in later samples. This is supported by the associations between isolation success and SARS-CoV-2 RNA copy numbers determined by quantitative PCR assay [38, 40]. For example, SARS-CoV-2 was isolated in all samples with *C*_t_ values in the range 13–17, but rarely (12 %) in samples with *C*_t_ values >30 [40]. Reports of virus isolation 20 days after symptom onset in individuals with severe COVID-19 and higher viral loads [41, 42] support this conclusion. Secondly, virus infectivity in *in vitro* culture may be neutralized by IgG or IgA after antibody seroconversion in the patient, even though the sample may contain intact virus particles. Finally, a broader comparison with other respiratory viruses is informative – if long-term persistence of viral and cellular debris accounted for long-term detection of SARS-CoV-2 RNA by PCR, then why would this not also occur in IAV and RSV infections, where the viral loads in acute infections are comparable to those in SARS-CoV-2?

There is increasing evidence for widespread systemic infection at extra-pulmonary sites by SARS-CoV-2, such as the GI tract, heart, kidneys and central nervous system (reviewed in [43]) and for its potential persistence at these sites after resolution of COVID-19 where it is possible to sample them (e.g. [27]). This naturally leads to the further question of whether such multi-system infection might underlie the often severe and diverse symptoms in ‘long COVID’. A proportion of individuals may experience many of the symptoms of chronic cough, shortness of breath, chest tightness, skin rashes, protracted loss or change of smell and taste, gastrointestinal disturbance with diarrhoea, continuing headaches, fatigue, weakness and sleeplessness, depression, anxiety and cognitive difficulties. It is clear, however, that much of the disease underlying these symptoms may originate from effects of lung scarring arising from the often severe and dysregulated cellular infiltration, hypercoagulation and pulmonary embolism in lung tissue during COVID-19 [44], and related inflammatory and thrombotic disease pathologies in other organs (reviewed in [45]). A recent study has, however, documented the presence of SARS-CoV-2 RNA and expressed viral proteins in the olfactory neuroepithelium in patients 110–196 days after COVID-19 [46]. The four study subjects reported persistent or intermittent loss of smell and taste dysfunction that would be consistent with ongoing replication of SARS-CoV-2 and associated inflammatory responses in this tissue. In a separate study of intestinal biopsies post-COVID-19, there was clear immunocytochemical evidence for SARS-CoV-2 replication in a high proportion of individuals [27, 47], although the sites of replication showed no overt cytopathology on histology analysis.

A related question is whether detection of SARS-CoV-2 in other sample types is also associated with transmissibility. Faecal samples are frequently SARS-CoV-2-positive in COVID-19 patients [32], from which positive virus cultures have been obtained, albeit infrequently [47, 48]. In addition to the evidence for active infection in regions of the GI tract that express the ACE-2 SARS-CoV-2 receptor [27, 47], SARS-CoV-2 may additionally target proximal tubule cells in the kidney that also express this receptor. However, urinary excretion of SARS-CoV-2 is rare (3–4 %; reviewed in [43]) and there are few reports of virus isolation of SARS-CoV-2 from this sample type [49]. Overall, and despite the widespread systemic infection and persistence in multiple organs [43], current evidence indicates that the infectivity and transmissibility of SARS-CoV-2 are confined to respiratory routes at least in the early stages of infection.

### Persistent infections with other coronaviruses

While the potential of SARS-CoV-2 to establish prolonged and potentially long-term persistent infections is a novel concept to most working in the COVID-19 area, even a cursory review of infections in other hosts reveals the long recognized capacity of a large number of other coronaviruses to establish long-term infections in birds, bats, rodents and domestic and companion animals [50]. These include bovine coronavirus [51, 52], mouse hepatitis virus and infectious bronchitis virus in birds [53, 54]. Pigs are infected with a range of different coronaviruses of variable propensities to establish persistent infections [55–58]. Cats infected with feline coronavirus (FCoV) similarly show high frequencies of prolonged, often lifelong, faecal shedding that maintains endemic transmission [59–63]. FCoV has been detected by PCR or virus isolation in around half of healthy cats in catteries, shelters or private households in cross-sectional studies [62, 64–66]. High detection rates in faecal samples from bats are similarly consistent with persistent infection by a range of coronaviruses in several species [67, 68]. Middle East respiratory syndrome coronavirus (MERS-CoV) RNA has been detected in over 40 % of animals in several groups of dromedary camels, which is also indicative of persistence [69] despite its more frequent although variable clearance in infected humans [70].

It has been generally considered that the human seasonal coronaviruses (HCoV-OC43, -NL63, -HKU1 and −229E) typically cause acute, mildly symptomatic respiratory infections. However, their potential to establish persistence has rarely been investigated using sensitive molecular detection methods. One recent study where individuals with diagnosed HCoV-NL63 were retested by PCR after 30–40 days indicated persistence or reactivation in 21 % of study subjects [71], findings remarkably similar to those observed for SARS-CoV-2 in the studies reviewed above.

### Genomic features of SARS-CoV-2

One prominent feature of SARS-CoV-2 and of all other coronaviruses infecting mammals and birds is the presence of large scale, pervasive RNA secondary structure throughout the genome [28]. Although a subset of structures relate to transcriptional- or replication-associated functions, such as the frameshift site in ORF1a/ORF1b, the wider distribution of RNA folding throughout the genome [28, 72] may correspond to the previously described genome-scale ordered RNA structure (GORS) in positive-stranded mammalian and avian RNA viruses. Possession of GORS is strongly associated with host persistence, although the mechanism(s) behind this association are largely uncharacterized [73, 74]. Genomes of coronaviruses are indeed typically even more intensely structured than those of persistent viruses characterized to date; SARS-CoV-2 genome sequences show a mean folding energy difference (MFED) of 15.1 % across the genome, compared to 8–9 % in different genotypes of hepatitis C virus (HCV) and 11 % in human pegivirus [73, 74]. Seasonal and newly emerging human coronavirus MFED values range from 8 % (HKU1) to 18 % (HCoV-OC43).

Even though functionally uncharacterized, the existence of shared genomic properties of SARS-CoV-2 and other coronaviruses with other persistent RNA viruses potentially provides insights into the nature of host interactions and immune responses. Among structured viruses, the best characterized is HCV, with a propensity to establish long-term sub-clinical infections and an associated, poorly understood failure of the T and B cell response to clear virus or generate protective immunity [75, 76]. The marked expression of pro-inflammatory cytokines IL-6 and IL-1β and apoptosis of reticulodendritic cells in SARS-CoV-2 infections [77, 78] show some parallels with the dysregulation of pro-inflammatory and regulatory cytokines that contribute to HCV-induced inflammatory disease [79, 80]. As with HCV, SARS-CoV-2 infections are associated with a range of functional defects of T and B cells that may contribute to its immunopathology and prolonged course of infection [29, 30, 78, 81] and the lack of durable antibody responses [82]. The recent demonstration of host genetic factors in cats that influence shedding durations and persistence of FCoV may indeed have parallels in SARS-CoV-2 infection [83]. The association with the *NCR1* gene encoding NKp46 is of particular interest, given its expression on natural killer (NK) cells and its role with Fc receptor γ in coupling innate immune responses to T cell regulation [84, 85]. This may be particularly relevant to infection outcomes for coronaviruses, HCV and other potentially persistent viruses, given the direct role of this regulatory pathway in determining acute or chronic outcomes in the widely used lymphocytic choriomeningitis virus infection/mouse model and the occurrence of T cell anergy [86]. This may be one of many pathways that couple interferon-associated innate cellular responses to viral RNA, an interaction potentially modulated by GORS, to the downstream adaptive immune responses to infection.

To conclude, the growing evidence for prolonged and often systemic SARS-CoV-2 infections, together with the virus’s shared genome structural features with persistent RNA viruses, caution against these initial assumptions of an acute resolving course of COVID-19 and the development of protective immunity to the virus. A schematic summary comparison of the various clinical and immunological features of SARS-CoV-2 with viruses causing acute and chronic infections, typified by IAV and HCV, respectively, indeed reveals attributes that are intermediate between the two (Table 1). Perhaps its recent zoonotic spread into humans from bats has not yet fully equipped SARS-CoV-2 for the fully persistent lifecycle observed in other coronaviruses. This may conceivably develop over time; longer-term shedding, along with enhanced infectivity and reduced pathogenicity, are potent evolutionary drivers underpinning the long-term survival of a virus in a new host. The D614G mutation in the spike protein that enhances entry [87] may be the first of many adaptive mutations in SARS-CoV-2, potentially analogous to those in the receptor-binding domain of the spike protein in severe acute respiratory syndrome coronavirus (SARS-CoV) and MERS-CoV [88, 89].

**Table 1.** Comparison of the infection and host response characteristics in SARS-CoV-2 infections with those of typical acute and persistent virus infections

	Acute infection Influenza A Virus	SARS-CoV-2	Persistent infection Hepatitis C virus
*Infection kinetics*	Short latent period, acute, resolving infections Short duration of shedding Immunopathology associated with virus clearance	Acute infections but often with prolonged virus shedding Frequent recurrence Unknown frequency of long-term carriage	Long latent period, acute infection, followed by delayed and often partial clearance Long-term persistence develops in over 60% of cases
*Immunity*	Potent cytotoxic T cell response Generation of durable protective neutralizing antibodies	Strong T cell response Variable and non-durable neutralizing antibody response Anergy and lymphopenia in severe COVID-19	T cells primarily responsible for initial control of virus replication Become anergic post-infection Neutralizing antibodies weak and ineffective
*Re-infection*	Long-term resistance to reinfection with the same serotype. Drives antigenic change	Reinfection increasingly recognized Non-durable NAb response may enable recurrence/reinfection	No immunity from reinfection after either spontaneous or treatment-induced virus clearance
*Transmission*	Acute phase only	No evidence for transmission after 8–10 days Infectivity during persistence, recurrence or reinfection unknown	Transmission occurs during both acute and persistent infection stages

Documenting the true nature of SARS-CoV-2 infections is clearly of vital importance in understanding its pathogenesis and treatment and in the more effective use of diagnostic testing algorithms and clinical follow-up. Most urgently, the assumption that disease resolution and the appearance of anti-SARS-CoV-2 IgG antibodies represent the resolution of infection should be rigorously evaluated.
